# Frequency and risk factors for recurrent gestational diabetes mellitus in primiparous women: a case control study

**DOI:** 10.1186/s12902-019-0349-4

**Published:** 2019-02-15

**Authors:** Yin-Yu Wang, Ye Liu, Cheng Li, Jing Lin, Xin-Mei Liu, Jian-Zhong Sheng, He-Feng Huang

**Affiliations:** 1 The International Peace Maternity and Child Health Hospital, Shanghai Jiao Tong University School of Medicine, No. 1961, Huashan Road, Shanghai, 200030 China; 2 The Key Laboratory of Reproductive Genetics (Ministry of Education), Women’s Hospital School of Medicine Zhejiang University, Hangzhou, 310058 People’s Republic of China; 3 Department of Pathophysiology, Zhejiang University School of Medicine, Hangzhou, 310058 People’s Republic of China

**Keywords:** Gestational diabetes mellitus, Recurrence, Risk factor, Biochemical parameter, Chinese primiparous woman

## Abstract

**Background:**

To investigate the frequency and risk factors for recurrent gestational diabetes mellitus (GDM) in Chinese primiparous women.

**Methods:**

Case control study. We investigated primiparous women who experienced GDM complications and had a subsequent pregnancy in the same hospital from January, 2012 to January, 2017. Ultimately, 78 women with recurrent GDM and 64 women with no recurrence were included. Clinical characteristics and biochemical parameters such as fasting plasma glucose (FPG), oral glucose tolerance test (OGTT) and lipid profiles were collected from medical records. We used an independent *t*-test and Chi-square test or Fisher’s exact test to compare each variable. Univariate and multivariate logistic analyses were used to compute each odds ratio (OR) and 95% confidence interval (CI).

**Results:**

The frequency of recurrent GDM was 55%. We found postprandial 1-h glucose at the 75-g OGTT was positively related to GDM recurrence, whereas first-trimester FPG in first pregnancy was negatively related. The first-trimester HbA1c value was higher in the group with GDM recurrence than in the group with no recurrence, though the difference was not significant. Moreover, the group with GDM recurrence manifested significantly higher first-trimester triglyceride concentrations in subsequent pregnancies; the adjusted ORs (95% CI) were 1.43 (1.09–1.87), 0.24 (0.10–0.63), 3.59 (0.93–13.88) and 1.89 (1.13–3.16).

**Conclusions:**

GDM recurred in more than half of subsequent pregnancies. Women with lower first-trimester FPG and higher postprandial 1-h glucose in first pregnancy, and with higher first-trimester triglyceride in subsequent pregnancy were at increased risk for GDM recurrence.

## Background

Gestational diabetes mellitus (GDM) is a common complication in pregnant women and is defined as maternal carbohydrate intolerance first diagnosed during pregnancy [1]. It was reported that the incidence of GDM was 4.3% in 2009 [2], and it increased to 17.5% in 2015 in Chinese women [3]; the prevalence has kept rising due to a great number of women living a less physically active lifestyle and entering pregnancy as overweight or obese [4, 5]. GDM is related to short-term and long-term adverse outcomes for both mother and offspring [6], such as pregnancy-induced hypertension, high rate of cesarean delivery, macrosomia, elevated risk of recurrence in subsequent pregnancy [7] and development of diabetes later in life [8, 9].

The reported frequency of GDM recurrence ranges widely from 30 to 84% [10], depending on the different populations studied and the various diagnostic criteria employed [11]. Although two studies reported the recurrence rates of GDM in Asia-Pacific countries [12, 13], limited data were correlated to the Chinese population [11, 14] because of the implementation of the “one-child policy” in China. Moreover, other studies [15, 16] reported significant differences in GDM incidence among different Asian countries and suggested that it is important to examine Asian subgroups separately. Risk factors related to GDM recurrence also have varied across previous studies. Of note, ethnicity was consistently associated with recurrence of GDM [10, 11]. In addition, advanced maternal age, pregestational obesity, insulin required in the first pregnancy, family history of diabetes in first-degree relatives, weight gain between pregnancies and postprandial glucose value at the oral glucose tolerance test (OGTT) were also strongly correlated with GDM recurrence [17]. However, other biochemical parameters such as triglycerides and fasting plasma glucose (FPG) during early pregnancy have not been widely evaluated as risk factors of GDM recurrence. In this study, we used a population of women delivering two sequential live singleton infants to examine the recurrence of GDM between parity one and parity two pregnancies.

Taken together, we conducted a case-control study to determine the frequency and risk factors associated with GDM recurrence in Chinese primiparous women, and documented first-trimester biochemical parameters. Our present study aimed to identify additional modifiable risk factors and help these high-risk women reduce the side effects that accompany subsequent GDM.

## Methods

### Patients

The subjects in the present study were all women with GDM between January, 2012 to June, 2015 and had a subsequent pregnancy between May, 2014 to January, 2017 at the International Peace Maternity and Child Health Hospital (IPMCH), Shanghai, China. Women with preexisting diabetes before pregnancy were excluded from this analysis. Women were divided into two groups based on the results of the 75-g OGTT in the subsequent pregnancy: the recurrence group and the group with no recurrence.

### Diagnostic criteria

The diagnostic criteria of GDM were made according to the International Association of the Diabetes and Pregnancy Study Group (IADPSG) recommendations, which are widely applied in the hospitals of China. Briefly, all pregnant women were screened for GDM using the one-step 2-h 75-g OGTT. Women were diagnosed as having GDM once glucose values met or exceeded the diagnostic criteria: fasting glucose value ≥5.1 mmol/L, 1-h glucose value ≥10.0 mmol/L, and 2-h glucose value ≥8.5 mmol/L.

### Investigation content

The following information was collected from medical records for each patient: maternal age, height, prepregnancy weight and body mass index (BMI), gestational weight gain, educational attainment (≤12, 13–16, or ≥ 17 years of completed schooling), family history of diabetes in the first-degree relatives, insulin treatment, mode of conception, mode of delivery, infant sex and weight, weight change between pregnancies, interpregnancy intervals, as well as biochemical parameters including FPG, uric acid, lipid profiles, glycated hemoglobin (HbA1c) and glucose values at the 75-g OGTT. All metabolic variables were measured at the first-trimester of pregnancy (12.0–14.0 gestational weeks) with the exception of the OGTT test and HbA1c in the first pregnancy, which were measured at a mean of 26.5 gestational weeks. BMI was calculated as weight in kilograms divided by height in meters squared. Weight change between pregnancies was referred to the change in prepregnancy weight between the first and subsequent pregnancies. The interpregnancy intervals, defined as the number of months between the date of delivery of the first pregnancy and the date of last menstrual period of the subsequent pregnancy, was also calculated.

### Statistical analysis

The data analyses were performed using SPSS 23.0 software (IBM SPSS, Chicago, IL, USA), and all variables were first assessed by normal distribution test (one-sample Kolmogorov-Smirnov test). If the data were normally distributed, we used an independent samples *t*-test for the continuous variables analysis. Dichotomous variables were evaluated with Chi-square test or Fisher’s exact test (where 20% or more of the cells in a χ2 table will have an expected count less than 5). Univariate logistic regression analysis was used to compute each odds ratio (OR) and 95% confidence interval (CI) and multivariate logistic regression analysis was used to identify independent predictors of GDM recurrence. Data are presented as the mean ± standard error (SE) or number (percentage) for normally distributed data. A two-sided *p* value less than 0.05 was considered statistically significant.

## Results

### Frequency

We collected the records of 142 primiparous women diagnosed with GDM in the first pregnancy; a total of 78 (55%) women had recurrent GDM in subsequent pregnancy.

### Maternal characteristics in the first pregnancy

Maternal characteristics in the first pregnancy are shown in Table 1. Demographic characteristics such as maternal age, prepregnancy weight and BMI, gestational weight gain, educational attainment, mode of conception and delivery, birth weight and the infant’s sex were comparable between the two groups. Although numbers were too small to detect potential significant differences, the proportions of family history of diabetes in first-degree relatives and insulin treatment were greater in the the group with GDM recurrence than the group with no recurrence. Biochemical parameters such as uric acid and cholesterol in the first-trimester, as well as fasting and postprandial 2-h glucose value at the OGTT test were also similar between the two groups. However, first-trimester FPG of the group with GDM recurrence was apparently lower, whereas triglyceride concentrations, postprandial 1-h glucose value and second-trimester HbA1c were significantly higher than the group with no recurrence.

**Table 1:** Maternal characteristics in the first pregnancy (*N* = 142)

Variables	Recurrent GDM *N* = 78	Primary GDM *N* = 64	Unadjusted OR (95% CI)	*p* value
Age (years)	30.0 ± 0.3	29.7 ± 0.4	1.05 (0.93–1.17)	0.442
Prepregnancy weight (kg)^a^	58.2 ± 1.2	58.8 ± 1.3	0.99 (0.95–1.04)	0.736
Prepregnancy BMI (kg/m^2^) ^a^	22.8 ± 0.5	22.2 ± 0.4	1.07 (0.94–1.22)	0.320
Gestational weight gain (kg) ^b^	11.7 ± 0.8	12.4 ± 0.6	0.97 (0.88–1.07)	0.510
Educational attainment (years)				
≤12	7 (9.0)	6 (9.4)	1.02 (0.32–3.23)	0.790
13–16	54 (69.2)	47 (73.4)	Ref	
≥17	17 (21.8)	11 (17.2)	1.35 (0.57–3.16)	
Family history of first-degree diabetes	20 (25.6)	11 (17.2)	1.66 (0.73–3.79)	0.228
Insulin treatment	4 (5.1)	1 (1.6)	3.41 (0.37–31.26)	0.279
Conception by ART	5 (6.4)	2 (3.1)	2.12 (0.40–11.33)	0.378
Delivery by Cesarean Section	32 (41.0)	23 (35.9)	1.24 (0.63–2.45)	0.536
Birth weight (g)	3264 ± 55	3300 ± 47	1.00 (0.99–1.01)	0.624
Male baby	34 (43.6)	27 (42.2)	1.06 (0.54–2.07)	0.867
Biochemical parameters in first-trimester (weeks)	13.3 ± 0.2	13.3 ± 0.3		
Fasting plasma glucose (mmol/L)	4.48 ± 0.05	4.66 ± 0.05	0.38 (0.17–0.88)	0.024^*^
Uric acid (umol/L)	226 ± 5	214 ± 5	1.01 (0.99–1.02)	0.108
Cholesterol (mmol/L)	5.01 ± 0.09	4.86 ± 0.11	1.28 (0.84–1.95)	0.250
Triglyceride (mmol/L)	1.68 ± 0.09	1.40 ± 0.07	1.94 (1.06–3.54)	0.031^*^
75-g OGTT in second-trimester (weeks)	26.4 ± 0.3	26.6 ± 0.4		
Fasting (mmol/L)	4.62 ± 0.07	4.51 ± 0.07	1.40 (0.77–2.52)	0.269
1 h (mmol/L)	10.44 ± 0.17	9.82 ± 0.18	1.34 (1.05–1.71)	0.018^*^
2 h (mmol/L)	8.67 ± 0.17	8.55 ± 0.20	1.05 (0.84–1.31)	0.653
HbA1c (%) ^c^	5.4 ± 0.1	5.3 ± 0.1	3.00 (1.03–8.78)	0.045^*^
[HbA1c (mmol/mol)]	36 ± 1	34 ± 1	1.11 (1.00–1.22)	0.045^*^

Variables are presented with mean ± standard error (SE) unless number (percentage)

*GDM* gestational diabetes mellitus, *OR* odds ratio, *CI* confidence interval, *BMI* body mass index, *ART* assisted reproductive technology, *OGTT* oral glucose tolerance test, *HbA1c* glycated hemoglobin

^a^ Prepregnancy weight and BMI: 32.4% missing;

^b^ Gestational weight gain: 41.5% missing;

^c^ HbA1c: 26.1% missing

### Maternal characteristics in the subsequent pregnancy

Maternal characteristics in the subsequent pregnancy are shown in Table 2. Demographic characteristics including maternal age, weight change between pregnancies and interpregnancy intervals showed no difference between the two groups, while the group with GDM recurrence manifested significantly higher prepregnancy BMI compared with the group with no recurrence. For biochemical parameters, there were not significant differences in first-trimester FPG, cholesterol, high-density lipoprotein and low-density lipoprotein cholesterol between the two groups. However, first-trimester HbA1c and triglyceride concentrations were significantly elevated in women who developed GDM in their subsequent pregnancy compared with women who did not.

**Table 2:** Maternal characteristics in the subsequent pregnancy (*N* = 142)

Variables	Recurrent GDM *N* = 78	Primary GDM *N* = 64	Unadjusted OR (95% CI)	*p* value
Age (years)	32.6 ± 0.3	31.9 ± 0.4	1.09 (0.97–1.21)	0.144
Prepregnancy weight (kg)	59.0 ± 1.2	57.2 ± 1.0	1.02 (0.99–1.06)	0.259
Prepregnancy BMI (kg/m^2^)	22.9 ± 0.4	21.7 ± 0.3	1.12 (1.00–1.25)	0.043^*^
Weight change between pregnancies (kg) ^a^	0.7 ± 0.7	−0.3 ± 0.7	1.04 (0.96–1.14)	0.340
Interpregnancy intervals (months)	22.6 ± 1.1	19.9 ± 1.3	1.03 (0.99–1.06)	0.135
Biochemical parameters in first-trimester (weeks)	12.7 ± 0.2	12.6 ± 0.2		
Fasting plasma glucose (mmol/L)	4.76 ± 0.07	4.61 ± 0.05	1.87 (0.93–3.74)	0.078
HbA1c (%)^b^	5.2 ± 0.0	5.1 ± 0.0	5.06 (1.45–17.64)	0.011^*^
HbA1c (mmol/mol)	34 ± 0	32 ± 0	1.16 (1.04–1.30)	0.011^*^
Cholesterol (mmol/L)	5.11 ± 0.11	5.00 ± 0.10	1.14 (0.79–1.64)	0.486
Triglyceride (mmol/L)	1.92 ± 0.10	1.53 ± 0.07	2.04 (1.23–3.36)	0.005^*^
HDL-C (mmol/L)	1.57 ± 0.04	1.70 ± 0.05	0.38 (0.14–1.01)	0.052
LDL-C (mmol/L)	3.28 ± 0.10	3.09 ± 0.10	1.35 (0.88–2.09)	0.172

Variables are presented with mean ± standard error (SE) unless number (percentage)

*GDM* gestational diabetes mellitus, *OR* odds ratio, *CI* confidence interval, *BMI* body mass index, *HbA1c* glycated hemoglobin, *HDL-C* high-density lipoprotein cholesterol, *LDL-C* low-density lipoprotein cholesterol

^a^ Weight change between pregnancies: 32.4% missing

^b^ HbA1c: 4.2% missing

### Independent risk factors

Potential risk factors were selected for inclusion in the multivariate logistic regression analysis to further examine the association with GDM recurrence. We included variables that were clinically important and/or statistically significant (*p* less than 0.05) between the two groups in the univariate logistic model. As shown in Table 3, the ORs were adjusted for maternal age and prepregnancy BMI in the first pregnancy, educational attainment, family history of diabetes, and insulin treatment. Notably, the first-trimester FPG and postprandial 1-h glucose value in the first pregnancy and first-trimester triglyceride concentrations in subsequent pregnancy were independent predictors of GDM recurrence.

**Table 3:** Selected characteristics in association with the risk for GDM recurrence (*N* = 142)

Variables	Adjusted OR ^a^ (95% CI)	*p* value
First-trimester fasting plasma glucose in the first pregnancy	0.24 (0.10–0.63)	0.004^*^
First-trimester triglyceride in the first pregnancy	1.77 (0.96–3.27)	0.069
1 h glucose in the first pregnancy (75-g OGTT)	1.43 (1.09–1.87)	0.010^*^
First-trimester HbA1c in subsequent pregnancy	3.59 (0.93–13.88)	0.063
First-trimester triglyceride in subsequent pregnancy	1.89 (1.13–3.16)	0.016^*^
First-trimester HDL-C in subsequent pregnancy	0.52 (0.19–1.44)	0.208

^a^ Adjusted for maternal age and prepregnancy BMI in subsequent pregnancy, educational attainment, family history of first-degree diabetes, and insulin treatment

*OR* odds ratio, *CI* confidence interval, *OGTT* oral glucose tolerance test, *HbA1c* glycated hemoglobin

## Discussion

This study demonstrated that the frequency of recurrent GDM in subsequent pregnancies was 55% of all women who developed GDM in their first pregnancy at IPMCH during the study period, which was in accordance with the pooled GDM recurrence among Asian women according to a recent meta-analysis [11]. However, the frequency from our study, which mainly involved Han Chinese, was higher than the frequency of 45% reported by Kwak et al. [12], whose population was predominantly Korean. One of the explanations for this different recurrence rate is that the underlying incidence of GDM in the Chinese population [2] is greater than the Korean population [16] and the higher incidence of GDM possibly indicates elevated susceptibility to its recurrence. Another explanation might rely on the different diagnostic criteria, since IADPSG criteria may double the number of GDM diagnosed women [18] and give rise to a sum of mild GDM patients [19]. In contrast, the frequency from our study was lower than the reports of Nohira et al. [13], whose frequency was 66%. Apart from the different diagnostic criteria, the Japanese study included both primiparous and multiparous women. Since primiparous women experienced a lower rate of GDM recurrence than multiparous women, the frequency will increased along with parity [11, 14, 19]. In addition, previous studies reported that the GDM frequencies ranged from 32 to 47% mainly based on Caucasian primiparous women [11, 20], which apparently lower than our results among Chinese primiparous women. This difference could be explained by ethnicity, as the frequency of GDM recurrence in Asians is higher than Caucasians [10, 11, 20]. Getahun et al. [21] suggested that large consumption of high glycemic index food and excess visceral adipose tissue accumulation might cause elevated levels of serum glucose and increase the risk of GDM among Asian populations. However, the women in our study had a mean prepregnancy BMI of 22.5 yet were susceptible to GDM despite not being overweight or obese. This fact highlights the difficulty in reducing the prevalence of GDM as more women developed western lifestyles associated with increasing wealth in China and globally.

Our findings were in agreement with previous studies [14, 19] which observed a positive association of high postprandial 1-h glucose value in the first pregnancy with GDM recurrence. Additionally, postprandial 1-h glucose can serve as a better predictor for development of type 2 diabetes mellitus (T2DM) compared with 2-h glucose [22, 23]. A probable reason is that the ability to utilize excess glucose in recurrent GDM patients decreases more severely than in primary GDM patients, which is similar to a study [23] that found that higher 1-h glucose indicated the existence of severe IR. Furthermore, since the OGTT directly reflects the degree of glucose tolerance and indicates the risk of developing T2DM [24], there is no doubt that the higher levels of OGTT results predict the greater likelihood of progression to GDM recurrence. This speculation is supported by higher HbA1c concentration in the second trimester, because the degree of IR is significantly related to the increase of HbA1c [25]. Thus, it would be acceptable to determine that the postprandial 1-h glucose of the OGTT in the first pregnancy is associated with GDM recurrence. Interestingly, first-trimester FPG in the first pregnancy was remarkably lower in the group with GDM recurrence than that in the group with no recurrence and the correlation with recurrence was stronger than postprandial 1-h glucose. It is widely accepted that a physiological reduction in FPG concentration occurs in normal pregnancy [26] and Zhu et al. [27] reported the median FPG among the Chinese population was 4.62 mmol/L at 12–14 gestational weeks. In our study, the median FPG was characterized by a lower level (4.50 mmol/L) in the group with GDM recurrence, whereas the level for the group with no recurrence (4.60 mmol/L) was comparable to the average. However, women in both groups had similar prepregnancy BMI with lower FPG in the group with GDM recurrence in the first pregnancy. Whereas, the women in the GDM recurrence group now have a higher prepregnancy BMI and the FPG slightly higher than the group with no recurrence in the second pregnancy. Moreover, the jump in TG levels between pregnancies is higher in the group with GDM recurrence than in the group with no recurrence. Although weight change between pregnancies is not different statistically because of significant missing data, it is conceivable from the above observations that women in the group with GDM recurrence gained more weight and adipose tissue than the other group making them prone to GDM despite their lower FPG in the first pregnancy. As a consequence, we suggest that the relation to FPG and GDM is likely to be a statistical relationship rather than a real mechanistic one.

One of the key features in our study was that we examined whether GDM recurrence was related to lipid profiles. It is of interest to mention that the first-trimester triglyceride concentration in subsequent pregnancy was significantly higher in the group with GDM recurrence, which agrees with the predictive value of the GDM occurrence [28] and progression to diabetes after GDM [29]. The triglyceride level was positively associated with the area under the postpartum glucose curve in women with prior GDM according to clinical research studies [30, 31]. These findings indicate that a common pathophysiological foundation underlies these diseases, and normalization of triglyceride level should be included as a preventive therapy. In addition, patients from both groups manifested elevated triglycerides in subsequent pregnancy, but the relative growth rates were 14.3 and 9.3%, for the GDM recurrence group and group with no recurrence, respectively. When circulating TG was at high levels, the formation of lipid droplets in the islet cells and the lipolysis of TG [32] were increased. On the one hand, the accumulation of TG causes cell toxicity and the number of islet cells decreases [33], which can aggravate IR and affect β-cell function. On the other hand, the risk of tissue exposure to free fatty acids (FFAs) increases, and high FFAs could result in IR via oxidative stress pathways [34]. That is, a higher triglyceride baseline in first pregnancy accompanied a greater increase in subsequent pregnancy, leading to double impairment for the group with GDM recurrence. Apart from the elevated serum triglycerides, a higher LDL-C (low-density lipoprotein cholesterol) level was observed in the group with GDM recurrence. A possible reason might correlate to the different nutrition structure and the amount of physical activity before subsequent pregnancy. Moses et al. [35] found that women with GDM recurrence had higher fat intake compared to those without recurrence according to their diet history and food records, which is consistent with our findings that higher LDL-C at the start of the subsequent pregnancy was a predictive factor for GDM recurrence. In addition, the group with GDM recurrence manifested lower HDL-C (high-density lipoprotein cholesterol) level than the group with no recurrence, which was also the strongest predictive factor of IR [36]. Therefore, early lipid profile derangement seems to have a critical impact on future glucose metabolism.

It is worth noting that we investigated risk factors for GDM recurrence among primiparous women in China. In addition, GDM recurrence patients were more likely to present higher prepregnancy BMI and lipid profiles such as LDL-C, which indicated that lifestyle intervention as soon as possible could reduce its recurrence.

Nevertheless, this study is not without limitations. First, the proportion of missing data was great including up to 32% of prepregnancy weights and 41% of gestational weight gains in first pregnancy, and approximately 32% of weight gains between pregnancies. Missing data were inevitable because the self-reporting of body weight was less accurate than measurement and records at the other hospitals were not always available, which may lead the weight change during consecutive pregnancies to become statistically insignificant between the two groups. Second, the missing data on the postpartum glucose tolerance test might have resulted in misdiagnosis of women with type 2 diabetes before the subsequent pregnancy and overestimated the GDM recurrence rate, which is also a common drawback in other studies [10, 11] due to general low compliance to conduct the postpartum diabetes screening. However, we strictly excluded patients suspected to have preexisting diabetes according to their medical records.

## Conclusions

In summary, GDM recurred in more than half of the women in their subsequent pregnancy among primiparous women in China. The association of postprandial 1-h glucose values in the OGTT with the recurrence of GDM was confirmed based on our study. In addition, decreased first-trimester FPG in the first pregnancy and the dyslipidemia profile identified in this sample of Chinese women appeared to be novel risk factors for the development of subsequent GDM. Together, early examination of metabolic risk factors could aid in assessing women who are at high risk for GDM recurrence.

## Abbreviations

BMI: Body mass index
CI: Confidence interval
FPG: Fasting plasma glucose
GDM: Gestational diabetes mellitus
HbA1c: Glycated haemoglobin
HDL-C: High-density lipoprotein cholesterol
IADPSG: International Association of the Diabetes and Pregnancy Study Group
IR: Insulin resistance
LDL-C: Low-density lipoprotein cholesterol
OGTT: Oral glucose tolerance test
OR: Odds ratio
T2DM: Type 2 diabetes mellitus
